# A Selective Culture System for Generating Terminal Deoxynucleotidyl Transferase-Positive Lymphoid Cells *In Vitro*. V. Detection of Stage-Specific Pro-B-Cell Stimulating Activity in Medium Conditioned by Mouse Bone Marrow Stromal Cells

**DOI:** 10.1155/1993/13675

**Published:** 1993

**Authors:** Sean D. Mckenna, Irving Goldschneider

**Affiliations:** Department of Pathology School of Medicine University of Connecticut Health Center Farmington Connecticut 06030 USA

**Keywords:** Terminal transferase (TdT), pro-B cell stimulating factor, *in vitro* culture system, bone marrow stromal cells

## Abstract

The selective *in vitro* generation of rat, mouse, and human terminal deoxynucleotidyl transferase-positive (TdT^+^ lymphoid cells in our long-term xenogeneic bone marrow (BM) culture system is characterized by physical interaction between the developing lymphocytes and mouse BM-adherent stromal cells and macrophages. In the present study, experiments in which micropor)us membrane culture inserts were inoculated with rat BM cells demonstrated that although the generation of primitive B-lineage lymphoid cells requires the presence of a mouse BM feeder layer, cognitive recognition events are not necessary. Similarly, cell-free (and serum-free) medium conditioned with mouse BM (but not thymus or spleen) adherent cells and stromal-cell lines therefrom supported the proliferation of early rat lymphoid cells in a dose-dependent manner. Double immunofluorescence for incorporated bromo-deoxyuridine (BrdU) and early B-lineage markers of rat BM lymphoid cells maintained in culture inserts or conditioned medium (CM), and studies of their in vitro and *in vivo* developmental potentials, indicated that the lymphoproliferative response resulted from the selective stimulation of lymphoid stem and/or progenitor cells. The most primitive of these target cells had a HIS24^+^ HIS50^-^ TdT^-^ cμ^-^ sIg^-^, pre-pro-B-cell phenotype. Whereas this subset normally constitutes less than 2% of B-lineage BM cells *in vivo*, it comprises more than 25% of total lymphoid cells *in vitro*. In addition, the number of TdT^+^ cells, predominantly of the early pro-B-cell phenotype (HIS24^+^ HIS50^-^ TdT^-^ cμ^-^ sIg^-^), was increased approximately tenfold above input levels. Based on these and previous findings, a schematic model is proposed for the developmental pathway of early B-lineage cells in rat BM from the level of the committed (possibly common) lymphoid stem cell to that of the pre-B-cell.


*Developmental Immunology*, 1993, Vol 3, pp. 181–195
Reprints available directly from the publisher
Photocopying permitted by license only

© 1993 Harwood Academic Publishers GmbH
Printed in the United States of America

### A Selective Culture System for Generating Terminal Deoxynucleotidyl Transferase-Positive Lymphoid Cells *In Vitro*. V. Detection of Stage-Specific Pro-B-Cell Stimulating Activity in Medium Conditioned by Mouse Bone Marrow Stromal Cells

SEAN D. McKENNA* and IRVING GOLDSCHNEIDER

*Department of Pathology, School of Medicine, University of Connecticut Health Center, Farmington, Connecticut 06030*

The selective *in vitro* generation of rat, mouse, and human terminal deoxynucleotidyl transferase-positive ($TdT^+$) lymphoid cells in our long-term xenogeneic bone marrow (BM) culture system is characterized by physical interaction between the developing lymphocytes and mouse BM-adherent stromal cells and macrophages. In the present study, experiments in which microporous membrane culture inserts were inoculated with rat BM cells demonstrated that although the generation of primitive B-lineage lymphoid cells requires the presence of a mouse BM feeder layer, cognitive recognition events are not necessary. Similarly, cell-free (and serum-free) medium conditioned with mouse BM (but not thymus or spleen) adherent cells and stromal-cell lines therefrom supported the proliferation of early rat lymphoid cells in a dose-dependent manner. Double immunofluorescence for incorporated bromo-deoxyuridine (BrdU) and early B-lineage markers of rat BM lymphoid cells maintained in culture inserts or conditioned medium (CM), and studies of their *in vitro* and *in vivo* developmental potentials, indicated that the lymphoproliferative response resulted from the selective stimulation of lymphoid stem and/or progenitor cells. The most primitive of these target cells had a $HIS24^+$ $HIS50^-$ $TdT^-$ $c\mu^-$ $sIg^-$, pre-pro-B-cell phenotype. Whereas this subset normally constitutes less than 2% of B-lineage BM cells *in vivo*, it comprises more than 25% of total lymphoid cells *in vitro*. In addition, the number of $TdT^+$ cells, predominantly of the early pro-B-cell phenotype ($HIS24^+$ $HIS50^-$ $c\mu^-$ $sIg^-$), was increased approximately tenfold above input levels. Based on these and previous findings, a schematic model is proposed for the developmental pathway of early B-lineage cells in rat BM from the level of the committed (possibly common) lymphoid stem cell to that of the pre-B-cell.

KEYWORDS: Terminal transferase (TdT), pro-B cell stimulating factor, *in vitro* culture system, bone marrow stromal cells.

### INTRODUCTION

The growth and differentiation of lymphoid cells from stem and progenitor cells are thought to be regulated by cell–cell contact as well as by the release of soluble factors (reviewed in Kincade et al., 1989; Dorshkind, 1990). A major technical advance in the study of these cellular and biochemical events in lymphopoiesis has been the development of long-term, feeder layer-dependent, *in vitro* bone marrow (BM) culture systems, in which manipulation of culture conditions profoundly affects the lineages and/or stages of development that are generated. For example, Dexter-type cultures selectively produce myeloid lineage cells and multipotential stem cells, but few lymphoid cells (Dexter et al., 1977), whereas the Whitlock–Witte culture system selectively produces large numbers of pre-B cells ($c\mu^+$, $sIg^-$), but not myeloid or multipotent stem cells (Whitlock and Witte, 1982; Whitlock et al., 1984). Recently, modifications to the Whitlock–Witte culture system have been described that permit the generation of B-lineage cells more primitive than those observed in standard cultures. These

*Corresponding author.

modifications include seeding the BM feeder layers with mouse fetal liver cells (Denis et al., 1984, 1987), transfecting the cultured ells with the BCR/ABL chimeric oncogene (Scherele et al., 1990), and initiating the culture in the presence of interleukin-4 (Peschel et al., 1989).

The role of soluble factors in the generation of pre-B-cells has been extensively studied using bone marrow stromal cell lines established from the foregoing cultures. In particular, interleukin-7 (IL-7), a cytokine first purified from mouse BM stromal cells, has been found to stimulate the proliferation of pre-B ($B220^+$) and possibly pro-B ($B220^-$) cells from mouse BM (Namen et al., 1988a,1988b; Henney, 1989; Lee et al., 1989). However, despite the effect of IL-7, the long-term survival of early B-lineage cells in primary cultures generally is not maintained in the absence of direct contact with BM stromal cells (Kierney and Dorshkind, 1987; Sudo et al., 1989). This is consistent with the development of foci of proliferating lymphoid precursor cells on and within the adherent BM stromal-cell layer (Whitlock and Witte, 1982; Whitlock et al., 1984; Hayashi et al., 1984; Medlock et al., 1993a, 1993b).

Hardy et al. (1991) have reported that the earliest phenotypically distinct mouse B-lineage cell population, called "pre-pro-" B-cells ($B220^+$, $S7^+$, $BP\text{-}1^-$, $HSA^-$, Ig genes in germline configuration), requires only contact with BM stromal cells to survive in a 4-day coculture system. However, later stages of lymphopoiesis, marked by expression of HSA and progressive Ig gene rearrangements, were increasingly feeder-layer adherence-independent and IL-7-dependent (Nishikawa et al., 1988; Sudo et al., 1989; Hardy et al., 1991). These observations suggest that close-range molecular interactions between lymphoid progenitor cells and microenvironmental cells regulate the initial stages of lymphopoiesis in BM. Although the nature of the requirement for close lymphoid-precursor:stromal-cell association is not known, a potential candidate for a stromal cell-bound second signal is the recently described stem-cell factor (SCF), which can act synergistically with IL-7 to stimulate lymphopoiesis (McNiece et al., 1991; Billips et al., 1992).

To specifically study the regulatory mechanisms that are operational at the most primitive stages of lymphoid development, we have designed a long-term xenogeneic BM culture system that selectively supports the generation of normal (and leukemic) lymphoid precursor cells from adult rat, mouse, and human BM on a mouse BM-adherent cell-feeder layer (Hayashi et al., 1984; Medlock et al., 1987a; Goldschneider and King, 1991; Medlock et al., 1993a, 1993b). The least mature lymphoid cells in the cultures of rat BM have a $HIS24^+$ $HIS50^-$ antigenic phenotype ($B220^+$ $HSA^-$ equivalent), lack the enzyme terminal deoxynucleotidyl transferase (TdT), and are mostly adherent to the mouse BM feeder layer. In contrast, the most mature cells in these cultures are $HIS24^+$ $HIS50^+$ $c\mu^-$ late pro-B-cells, and are present primarily in the nonadherent phase of the culture.

Despite these observations, it is not clear that adherence to the feeder layers is essential for the maintenance of lymphopoiesis in our culture system. Thus, although lymphoid precursor cell activity is approximately tenfold higher per unit number of cells in the adherent than in the nonadherent phase of the culture (Medlock et al., 1993b), approximately 20% of the nonadherent lymphoid cells are in DNA synthesis (Hayashi et al., 1984). Moreover, reduction of the concentration of fetal bovine serum (FBS) in the culture medium markedly decreases the percentage of adherent $TdT^+$ lymphoid cells, but not the total number of lymphoid cells generated (Medlock et al., 1993b). Therefore, the requirement for physical contact between the developing lymphoid cells and the mouse BM-adherent cells was formally investigated in the present study. The results demonstrate that rat BM $TdT^+$ pro-B-cells and their $TdT^-$ precursors (pre-pro-B-cells) can be generated both in microporous membrane culture inserts placed over mouse BM-adherent cell-feeder layers, as well as in serum-free medium conditioned by stromal cells from these feeder layers. Hence, although contact between these primitive lymphoid precursors and mouse BM stromal cells may optimize lymphopoiesis in our culture system, the generation of pro-B-cells is, in the final analysis, maintained by a soluble factor(s) from such stromal cells.

### RESULTS

### Selective Generation of Rat BM Lymphoid Precursor Cells in Culture Inserts

To examine whether physical contact between the mouse BM-adherent feeder-layer cells and the rat BM lymphoid precursor cells is required for the generation of $TdT^+$ lymphoid cells *in vitro*, freshly harvested rat BM cells were cultured in microporous membrane culture inserts placed over (but not in contact with) confluent mouse BM feeder layers for 10 days. As demonstrated in Fig. 1A, rat BM lymphoid cells were selectively maintained in these culture inserts, but not in inserts placed in wells lacking a feeder layer. Typically, lymphoid cells accounted for 60–90% of total cells recovered from the culture inserts. Of these, approximately 30–50% were TdT$^+$, as compared with 3–5% of total cells in the starting inoculum.

Results in Fig. 1B show that lymphopoiesis could be maintained for at least 3 weeks by serially passaging the culture inserts onto fresh mouse BM feeder layers at 10-day intervals. However, when the culture inserts were transferred to wells containing rat (instead of mouse) BM feeder layers, or culture medium only, the lymphoid cells rapidly died. This is consistent with our earlier observation that rat BM feeder layers do not, by themselves, support BM lymphopoiesis *in vitro*, even when direct cell contact is permitted (Hayashi et al., 1984).

As in the standard culture system, only extremely immature B-lineage cells, almost all of which express the HIS24 marker (Opstelten et al., 1986), were maintained in the culture inserts (CI). Thus, as compared with the original BM-cell inoculum, day 10 CI-generated lymphoid cells were completely depleted of sIg$^+$ B cells and c$\mu^+$ pre-B-cells, and partially depleted (approximately twofold) of intermediate and late pro-B-cells (HIS50$^+$ TdT$^+$ and HIS50$^+$ TdT$^-$, respectively) (Table 1). Conversely, the CI-generated lymphoid cells were enriched approximately twofold for the HIS50$^-$ TdT$^-$ subset of pre-pro-B-cells, and tenfold for the HIS50$^-$ TdT$^+$ subset of early pro-B-cells, which together constituted 70% of the total lymphoid cells present. The developmental relationships of these phenotypic subsets have been established in previous experiments in which HIS24$^+$ HIS50$^-$ TdT$^-$ cells generated HIS24$^+$ HIS50$^-$ TdT$^+$ and thence HIS24$^+$ HIS50$^+$ TdT$^+$ and HIS24$^+$ HIS50$^+$ TdT$^-$ cells *in vitro* (Goldschneider et al., 1987; Hunt et al., 1988; Medlock et al., 1993b). Similarly, HIS24$^+$ HIS50$^-$ cells in normal rat BM are thought to be the immediate precursors of HIS24$^+$ HIS50$^+$ pro-B-cells *in vivo* (Hermans, 1991).

Although the proportions of rat TdT$^+$ and TdT$^-$ lymphoid cells generated in culture inserts was similar to that generated directly on mouse BM-adherent cell-feeder layers, the number of total lymphoid cells present was approximately tenfold lower. One possible explanation is that direct contact of the lymphoid precursor (or other) cells in rat BM with the mouse BM feeder-layer cells may increase the production and/or release of lymphostimulatory factors into the medium (Sudo et al., 1989). To address this possibility, rat BM cells were cultured for 10 days in microporous membrane inserts placed over mouse BM feeder layers that had also been seeded with rat BM cells. Although normal numbers of lymphoid cells were generated on the feeder layers, the number of lymphoid cells recovered from the culture inserts placed over the seeded feeder layers was approximately 25% lower (rather than higher) than that from inserts placed over unseeded feeder layers (data not shown).

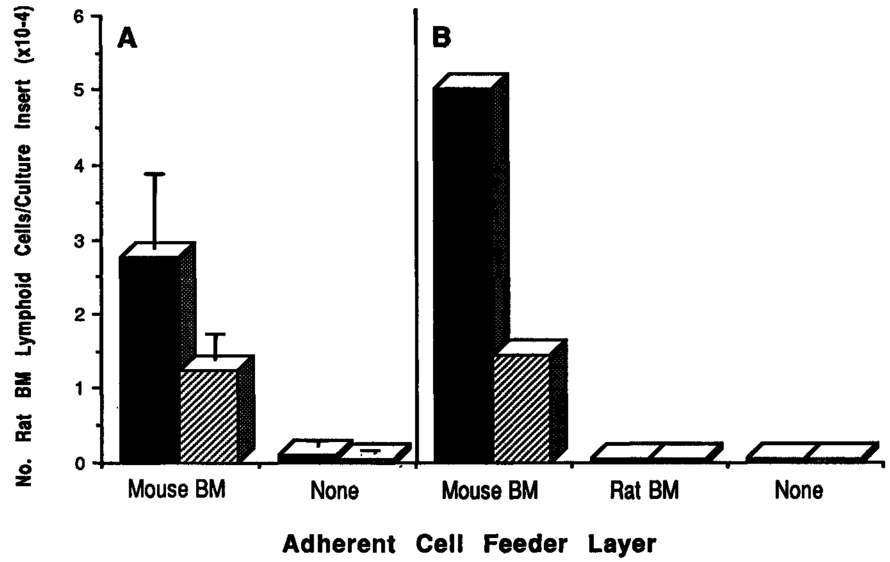

FIGURE 1. Maintenance of rat BM lymphoid precursor cells in culture inserts. $5\times10^5$ freshly harvested rat BM cells were added to microporous membrane culture inserts placed in wells in the presence or absence of a mouse BM-adherent cell-feeder layer. Results represent the mean number (±S.D.) of total (solid) and TdT$^+$ (hatched) lymphoid cells per culture insert: (A) 10 days later, and (B) 10 days after transfer of the culture inserts to new wells in the presence or absence of a mouse or rat BM-adherent cell-feeder layer (20 days total elapsed time *in vitro*). Similar numbers of TdT$^+$ lymphoid cells were maintained in culture inserts upon transfer to tertiary feeder layers (30 days total elapsed time).

TABLE 1
Fate of Early B-Lineage Cell Subsets Cultured in Microporous Membrane Inserts[a]

| Stage of development | | Number of HIS24+ Rat BM lymphoid cells/culture insert ($\times 10^{-4}$) | | |
|---|---|---|---|---|
| Subset | Phenotype | Input | Day 10 | Relative change |
| Total | — | 20 | 4.3 | -4.7-fold |
| B | sIg+ | 5 | <0.5 | -10-fold |
| Pre-B | $c\mu^+$ | 10 | <0.5 | -20-fold |
| Pro-B (late) | TdT− HIS50+ | 2.3 | 0.8 | -2.9-fold |
| Pro-B (interm) | TdT+ HIS50+ | 1.8 | 1 | -1.8-fold |
| Pro-B (early) | TdT+ HIS50− | <0.1 | 1 | +10-fold |
| Pre-pro-B | TdT− HIS50− | 0.8 | 1.5 | + 1.9-fold |

[a]Freshly harvested rat BM cells were placed in culture inserts ($5 \times 10^5$ cells/insert) over confluent mouse BM-adherent cell layers on day 0. Ten days later, cells were recovered for immunofluorecence analysis.

Another possible explanation for the greater generation of lymphoid cells in the standard culture system than in culture inserts is that some of the lymphostimulatory activity normally is bound to components of the extracellular matrix (ECM) and/or cell membranes in the feeder layer (Gordon et al., 1987; Roberts et al., 1988). To test this, mouse BM-adherent cell-feeder layers were extracted with 2M NaCl solution (Gordon et al., 1987), desalted by ultrafiltration (MW cutoff 10 kD) in serum-free culture medium, and brought up to 25% FBS. Although the saline solution did not affect the lymphostimulatory activity in medium conditioned by mouse BM feeder layers (see what follows), no lymphostimulatory activity was detected in the saline extract from these feeders (data not shown).

### Selective Generation of Rat BM TdT+ Lymphoid Cells in Conditioned Medium (CM)

To more directly examine the role of soluble factors in the generation of primitive lymphoid cells *in vitro*, freshly harvested rat BM cells were cultured in medium that had been conditioned for 10 days with mouse BM-adherent cell-feeder layers. After 8 days in the CM, there was a readily discernible increase in the percentage of rat TdT+ lymphoid cells over that in control medium. However, it was difficult to quantify this increase due to the presence of large numbers of dead myeloid cells. To circumvent this, enriched suspensions of rat BM lymphoid cells, generated in standard 10-day cultures, were substituted for freshly harvested rat BM cells in the CM. As shown in Figs. 2 and 3, the lymphoid cells were maintained for at least 4 days (>90% viability) by CM from mouse BM feeder layers, but not by CM from mouse thymic or splenic adherent cells or rat BM feeder layers. Moreover, approximately 50% of the accumulated lymphoid cells expressed TdT.

Results in Table 2 demonstrate that the rat TdT+ BM lymphoid cells recovered from CM after 4 days were phenotypically similar to those harvested from culture inserts and standard cultures, supporting the notion that pro-B-cells in these cultures are selectively stimulated by soluble mediators from the mouse BM-adherent cell feeder. This was further tested in experiments in which culture-generated rat BM lymphoid cells, incubated for 4 days in CM, were pulsed with BrdU. As shown in Table 3, approximately one-third of the lymphoid cells incorporated BrdU and all of these had the HIS24+ HIS50− phenotype. Conversely, approxi-

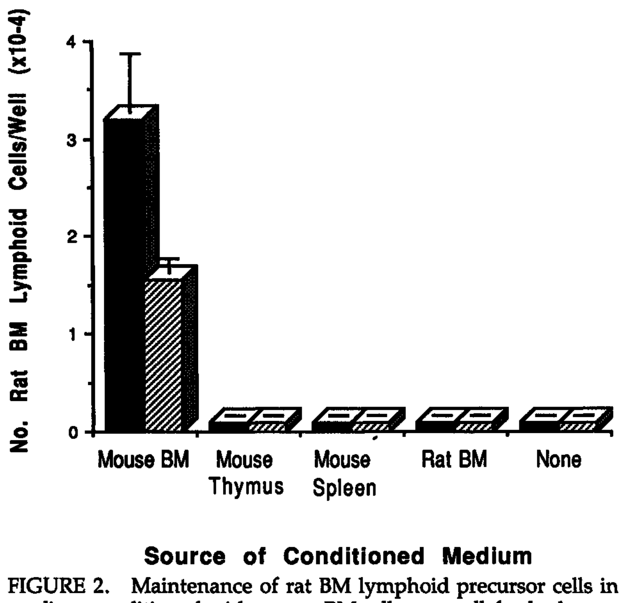

FIGURE 2. Maintenance of rat BM lymphoid precursor cells in medium conditioned with mouse BM-adherent cell-feeder layers. Medium was conditioned for 10 days by adherent cells from the indicated tissues and diluted twofold in nonconditioned medium. $5 \times 10^5$ culture-generated rat BM lymphoid cells were incubated for 4 days in 2 ml of CM. Results represent the mean number (±S.D.) of total (solid) and TdT+ (hatched) lymphoid cells per well.

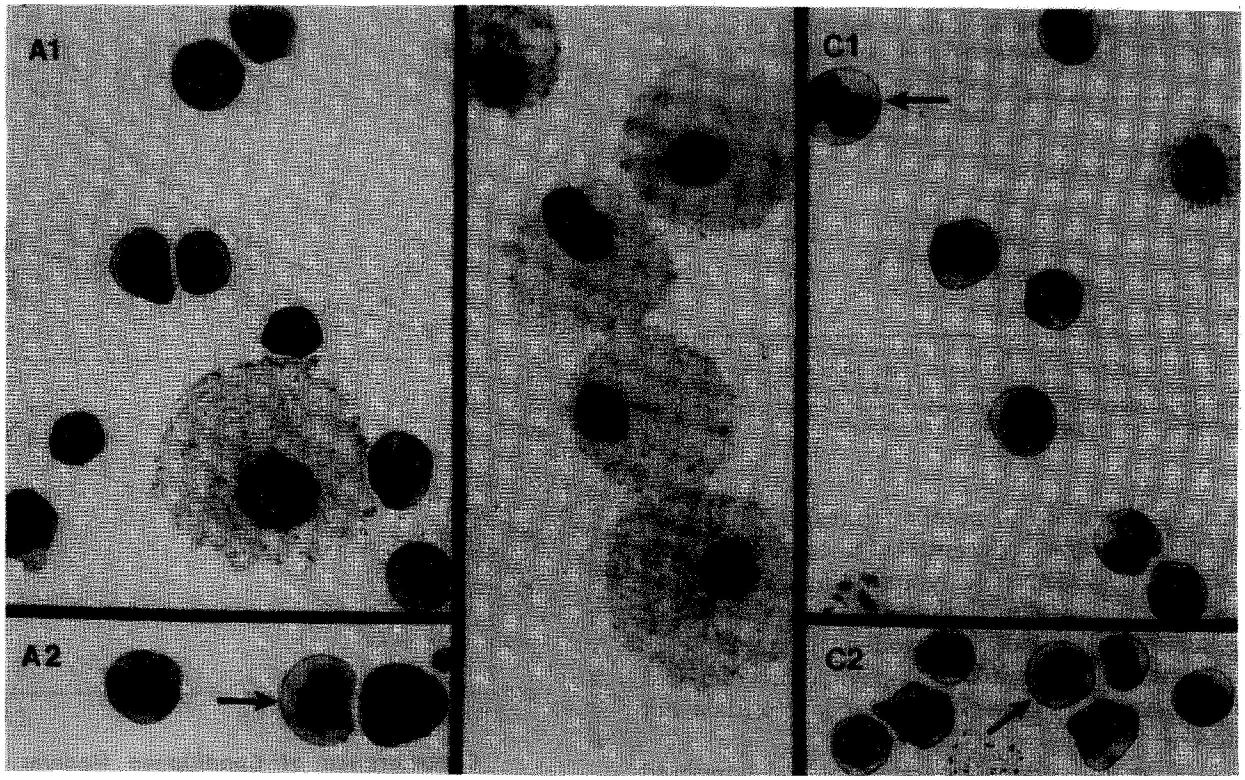

FIGURE 3. Morphology of rat BM cells generated in the absence of direct contact with mouse BM feeder layers. The lymphoid cells were originally generated for 10 days in microporous membrane culture inserts (A, B) or in standard cultures (C), and the lymphoid cells were cultured for an additional 4 days in medium conditioned by mouse BM-adherent cells (A, C) or in normal medium (B). Numerous immature lymphoid cells, some undergoing mitosis (arrow), and occasional macrophages are present in (A) and (C), whereas only macrophages are present in (B). May Grunwald-Giemsa stain. ×1000.

TABLE 2
Phenotype of Rat BM TdT$^+$ Lymphoid Cells Before and After Cultured *In Vitro*[a]

| | Percent positive cells | | | |
|---|---|---|---|---|
| Markers | Conditioned medium (day 4) | Culture insert (day 10) | Standard culture (day 10) | Fresh RBM |
| Rat MHC (RT-1$^l$) | >95 | >95 | >95 | >95 |
| Mouse MHC (H-2$^b$) | <1 | <1 | <1 | <1 |
| Surface Ig | <1 | <1 | <1 | <1 |
| Cytoplasmic $\mu$ | <1 | <1 | <1 | <1 |
| Ox-19 (Pan T cell) | <1 | <1 | <1 | <1 |
| Ox-39 (IL-2R) | <1 | <1 | <1 | <1 |
| HIS24 (CD45R-B220) | >95 | >95 | >95 | >95 |
| HIS50 (HSA) | 35 | 50 | 49 | 96 |

[a]Cells analyzed by double immunofluorescence for TdT and indicated markers were (1) culture-generated rat BM lymphoid precursor cells incubated in CM, (2) rat BM lymphoid precursor cells generated in culture inserts in the presence of mouse BM-adherent cell-feeder layer, (3) nonadherent rat lymphoid cells recovered from standard cultures, and (4) freshly harvested rat BM cells.

mately two-thirds of the HIS24$^+$ HIS50$^-$ cells incorporated BrdU, and approximately 50% of these were TdT$^+$. It should be noted that incubation with BrdU did not alter the total number of lymphoid cells recovered from the CM-treated cultures.

Dose-response experiments demonstrate that both the number of lymphoid cells recovered from the CM (Fig. 4A) and the incorporation of tritiated thymidine by these cells (Fig. 4B) was proportional to the concentration of CM after ultrafiltration, as

TABLE 3
Phenotype and BrdU Incorporation of Rat BM Lymphoid Cells Incubated in Medium Conditioned by Mouse BM-Adherent Cells[a]

| Markers | Percent marker-positive cells | Percent BrdU-positive cells |
|---|---|---|
| Total Lymphoid Cells | 100 | 35 |
| TdT+ | 50 | 44 |
| TdT- | 50 | 42 |
| HIS24+ | 100 | 39 |
| HIS24- | <1 | <1 |
| HIS50+ | 39 | <1 |
| HIS50- | 61 | 71 |

[a]Culture-generated rat BM lymphoid cells were incubated in twofold concentrated mouse BM-conditioned medium for 4 days. Twelve hours prior to harvesting, the cells were pulsed with BrdU. Incorporation of BrdU and expression of the indicated markers were detected by double immunofluorescence. No BrdU incorporation was observed in the surviving cells cultured in control medium.

was the frequency of mitotic figures in cell smears (Fig. 3). In contrast, concentrated unconditioned medium had no detectible lymphostimulatory effect. Furthermore, medium conditioned with mouse BM feeder layers that had been seeded with rat BM cells had *less* lymphostimulatory activity than did CM from unseeded mouse BM feeder layers (data not shown).

Because the culture-generated rat BM lymphoid cells in the preceding experiments might have been contaminated with mouse BM cells from the feeder layer, the ability of CM to support the growth of rat BM lymphoid cells, generated exclusively in culture inserts, was also tested. The results indicated that rat BM lymphoid cells' from both sources were maintained equally well by CM (Fig. 3 and data not shown). This strongly suggests that the CM stimulates early lymphopoiesis in rat BM by acting directly on the lymphoid precursors.

We next determined whether active CM could be generated in the absence of serum. Mouse BM feeder layers were intially cultured for 10 days in medium containing 25% FBS, after which they were washed extensively with and cultured for an additional 4 days in serum-free RPMI-1640. (The feeder layers could be maintained for 4 to 5 days in this serum-free medium before showing signs of deterioration.) The serum-free CM (SFCM) was then concentrated up to twentyfold by ultrafiltration and added to culture-generated rat BM lymphoid precursor cells in the presence of 25% FBS. As demonstrated in Fig. 5, the number of lymphoid cells obtained 4 days later was a function of the concentration of SFCM. It was further observed that lymphostimulatory activity comparable to that in ten-fold concentrated SFCM was obtained in unconcentrated day 10 CM gener-

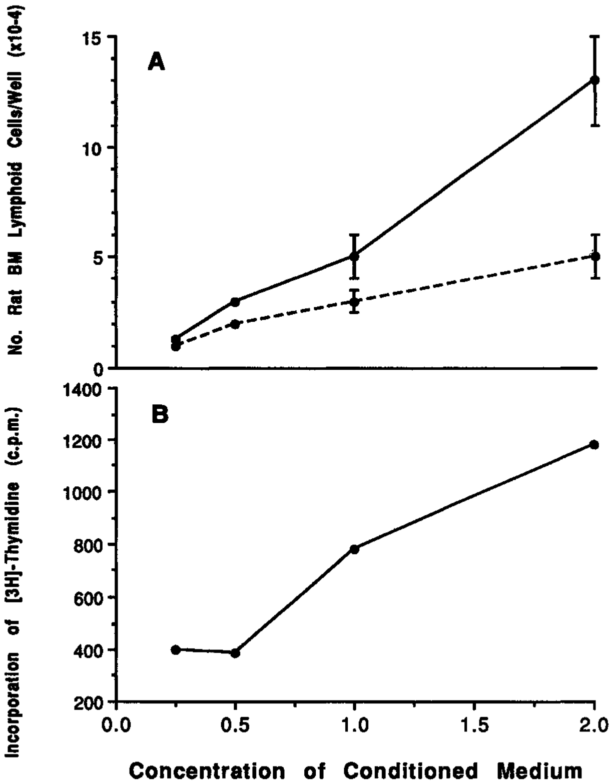

FIGURE 4. Number of rat BM lymphoid precursor cells (A) and incorporation of tritiated thymidine (B) as a function of the concentration of mouse BM-adherent cell-conditioned medium. CM was concentrated tenfold by ultrafiltration and diluted with nonconditioned medium to the indicated final concentrations, where 1 is equivalent to unconcentrated, undiluted CM. Units are given with respect to unconcentrated CM. (A) $5\times10^5$ culture-generated rat BM lymphoid cells were incubated for 4 days in 2 ml of CM. Results represent the mean (±S.D.) number of total (solid line) and TdT+ (dashed line) lymphoid cells per well. (B) $1\times10^5$ culture-generated rat BM lymphid cells were placed in 0.2 ml CM for 72 hr. Twelve hours prior to harvesting, wells were pulsed with 1 mCi[$^3$H]-TdR. Results represent c.p.m. of [$^3$H]-TdR incorporated by total lymphoid cells per well in a representative experiment (one of three).

ated in medium containing 1% FBS and 5% Nutridoma (data not shown).

### Developmental Potential of CM-Sensitive Rat Lymphoid Cells

As the lymphoid cells that were stimulated by CM appeared to have a very immature phenotype, it was of interest to determine whether any of these cells could function as lymphoid progenitors *in vitro* when replated in standard cultures. The results in Fig. 6 show that after incubating freshly harvested rat BM cells in CM for 4 days or culture inserts for

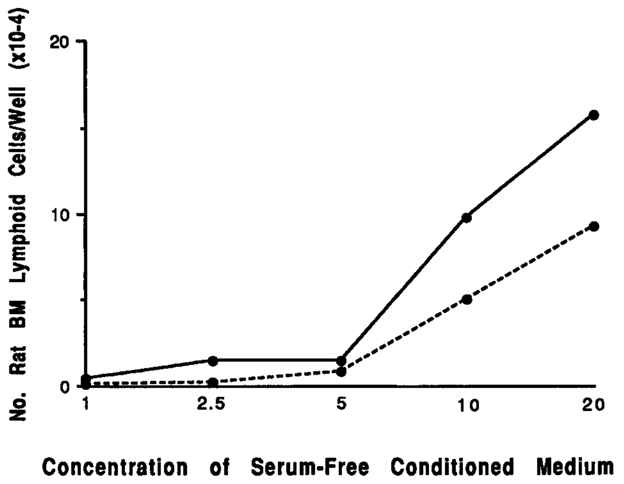

FIGURE 5. Maintenance of rat BM lymphoid precursor cells in various concentrations of serum-free conditioned medium. The SFCM, prepared as described in Methods, was collected from mouse BM-adherent cells after 4 days, concentrated twentyfold by ultrafiltration and diluted with serum-free medium to the indicated concentration, where 1 is equivalent to unconcentrated, undiluted SFCM. $5 \times 10^5$culture-generated rat BM lymphoid precursor cells were cultured for 4 days in 2 ml of SFCM after being supplemented with FBS to a final concentration of 25%. Results represent the number of total (solid line) and $TdT^+$ (dashed line) lymphoid cells per well.

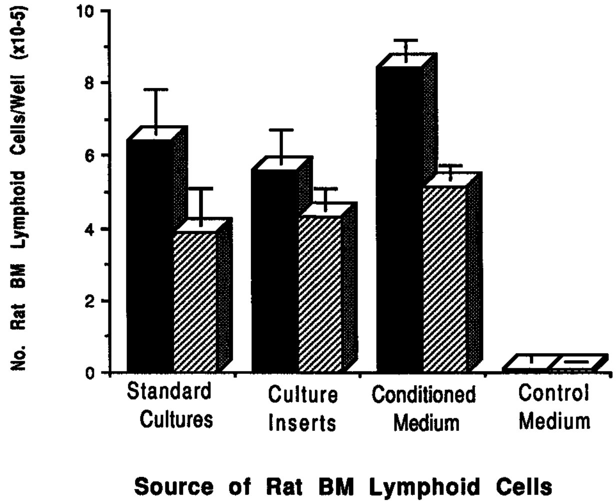

FIGURE 6. Lymphoid progenitor cell activity of rat BM cells maintained in culture inserts or conditioned medium. $1 \times 10^6$ freshly harvested rat BM cells were incubated for 4 days in medium conditioned with mouse BM adherent cells or in culture inserts placed over mouse BM-adherent cell-feeder layers for 10 days. The cells were then transferred directly onto confluent mouse BM feeder layers for an additional 10 days. Results represent the mean number (±S.D.) of total (solid) and $TdT^+$ (hatched) rat lymphoid cells recovered in the nonadherent phase of these secondary cultures, as compared with the number of lymphoid cells generated 10 days after seeding $1 \times 10^6$ freshly harvested rat BM cells directly onto mouse BM feeder layers.

10 days, progenitor activity approximately equivalent to that present in the nonadherent phase of standard cultures was recovered. These progenitor cells, after transfer to the feeder layers, formed lymphoproliferative foci on and within the mouse BM-adherent cells. Conversely, only minimal progenitor activity was detected in rat BM cells following 4 days of culture in control medium.

To test the *in vivo* lymphopoietic potential of the CM-sensitive BM cells, freshly harvested rat BM cells were cultured in CM for 4 days prior to being injected intravenously into sublethally irradiated, RT-7 and Igk-1 alloantigen disparate rats. After 25 days, the spleens and thymuses of the recipient rats were collected and stained with antibodies specific for donor B cells (anti-$Igk1^b$) and T cell (anti-$RT\text{-}7^b$). Unlike human and mouse B cells, approximately 95% of rat B cells express the $\kappa$ Ig light-chain isotype (Springer et al., 1982), so that it is a useful allogspecific marker for sIg+ B cells. As demonstrated in Fig. 7, rat BM cells precultured in CM, as those obtained from standard cultures, maintained a significantly higher capacity to regenerate both the B- and T- cell compartments in the recipient rats than those cells that were precultured in control medium. Conversely, the level of pluripotent stem-cell activity, as detected indirectly by CFU-S assay, decreased more than 90% during culture in both CM and control medium, and more than 95% in standard cultures, as shown previously (Hayashi et al., 1984).

### Constitutive Release of Lymphostimulatory Activity by Mouse BM Stromal-Cell Lines

Mouse BM-adherent cell-feeder layers were repeatedly passaged at 10-day intervals in order to generate stromal-cell lines. After approximately 2 months, two lines of adherent cell (F12-5B6 and F1-12B6), each having a homogenous stromal-cell morphology, were isolated. As shown in Fig. 8, these cell lines constitutively generated stimulatory activity for rat BM lymphoid precursor cells. However, after approximately 6 months of continuous activity, these cell lines lost the ability to spontaneously condition medium. This latter phenomenon, which subsequently has been observed with several other stromal cell lines, is not associated with obvious changes in proliferative activity or morphology of the stromal cells.

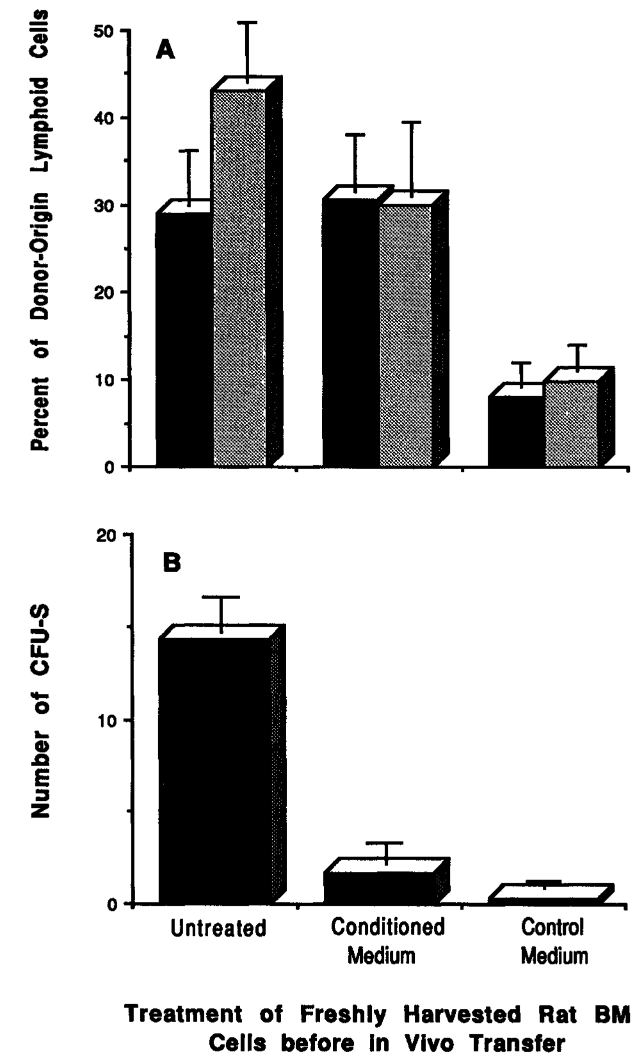

FIGURE 7. Mouse BM-adherent cell-conditioned medium maintains B- and T-lineage lymphoid progenitor cells. Freshly harvested rat BM cells were incubated for 4 days in medium conditioned for 10 days with mouse BM-adherent cells ($1\times10^6$ cells/ml). Sublethally irradiated rats were injected intravenously with $25\times10^6$ CM-treated cells for lymphocyte regeneration assay or $1\times10^6$ cells for CFU-S assay. Results in (A) represent the percentage (±S.D.) of donor-origin thymocytes (dark) and splenic B cells (light) 25 days postinjection, as determined by immunofluorescence staining with RT-7 and IgK-1 alloreactive antibodies, respectively. Results in (B) represent the mean number (± S.D.) of CFU-S per spleen 12 days postinjection.

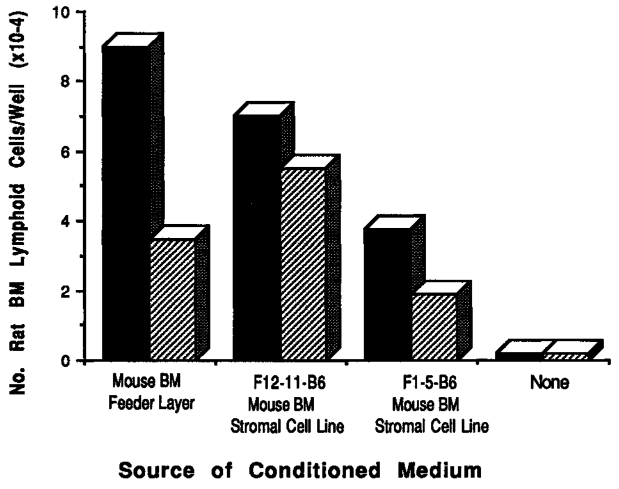

FIGURE 8. Lymphostimulatory activity in medium conditioned by mouse BM stromal cell lines. $5\times10^5$ culture-generated rat BM lyphoid cells were incubated for 4 days in 2 ml of the indicated CM. The mouse BM stromal cell lines (F12-11-B6, F1-5-B6) were produced as described in Methods. Results represent the number of total (solid) and TdT$^+$ (hatched) BM lymphoid cells per well. Similar levels of activity were detectable in CM from the respective stromal cell lines for up to 6 months. Representative experiment (one of five).

### DISCUSSION

Neither pre-B-cells nor their immediate progenitors are supported in culture inserts under Whitlock–Witte culture conditions (Kierney and Dorshkind, 1987). However, cells that can give rise to pre-B-cells when transferred directly onto Whitlock–Witte feeder layers can survive in culture inserts under Dexter-type culture conditions. Although the nature of these adherence-independent precursors is unknown, it is intriguing to speculate, given the results presented herein, that they may be related to the small number of TdT$^+$ lymphoid cells that we previously have observed in standard Dexter cultures (Schrader et al., 1978).

Stem-cell factor, a regulatory mediator produced by stromal cells in both a soluble (Williams et al., 1990; Zsebo et al., 1990) and membrane-bound (Flanagan and Leder, 1990; Huang et al., 1990) form, appears to act synergistically with lineage-specific factors to stimulate the most immature members of a variety of hemopoietic cell lineages and may be involved in the adherence-dependent stage of pre-B-cell generation. However, membrane-bound SCF does not appear to play a major (or essential) role in our culture system, inasmuch as BM-adherent cells derived from *Sl/Sl$^d$* mutant mice effectively support the generation of TdT$^+$ lymphoid cells *in vitro* (Medlock et al., 1987b). Moreover, the failure to recover lymphostimulatory activity from the feeder layer by extraction with hypertonic saline suggests, but does not prove, that early lymphopoiesis is not enhanced by other mediators that may be bound to cell membranes or ECM (Gordon et al., 1987; Roberts et al., 1988).

It therefore is of interest that decreasing the concentration of FBS in the culture medium markedly reduces the percentages of $TdT^+$ lymphoid cells in the adherent phase of the culture, but does not affect the total number of lymphoid cells that are generated (Medlock et al., 1993b). This observation permits two inferences: first, that a hyaluronate-dependent adhesion system, regulated by serum (Matuoka et al., 1987) and similar to that described in Whitock–Witte cultures (Miyake et al., 1990), may be operational in our culture system; and, second, this adhesion system is not essential for optimal lymphopoiesis under conditions in which $TdT^+$ lymphoid precursors can continue to contact the feeder layer. However, the lowered efficiency of early lymphopoiesis observed in culture inserts and in unconcentrated CM, and the continued adherence of most $TdT^-$ lymphoid cells under low serum conditions, leaves open the possibility that direct contact and/or adherence between at least a subset of lymphoid precursor cells and feeder-layer cells is important for *optimal* lymphopoiesis.

Some investigators have found that direct contact between lymphoid cells and stromal cells induces the stromal cells to increase the level of production of cytokines such as IL-7 (Sudo et al., 1989). We therefore determined whether seeding of rat lymphoid precursor cells directly onto the mouse BM feeder layer could increase the number of $TdT^+$ lymphoid cells generated in culture inserts or CM by a similar mechanism. The observation that the number of $TdT^+$ cells in the culture inserts and CM was *diminished* rather than increased under these conditions suggests that the effective concentration of relevant soluble mediator(s) available to lymphoid precursors in culture inserts was not increased, possibly due to their preferential usage by the lymphoid cells in closest proximity to the feeder layer. This in turn suggests that the reduced efficiency of lymphopoiesis observed among rat BM cells placed in culture inserts is due, at least in part, to a suboptimal concentration gradient of soluble mediators. The dose-response effects on the numbers of $TdT^+$ cells generated in CM further supports this notion.

As reported previously (Hayashi et al., 1984) and confirmed here, neither adherent cell feeder layers from mouse thymus or spleen nor CM therefrom support early lymphopoiesis in our culture system. This suggests that the lymphopoietic activity produced by mouse BM stromal cells in organ-specific. The failure of rat BM-adherent cell-feeder layers and CM therefrom to support lymphopoiesis *in vitro* does not contradict this thesis, inasmuch as rat BM-adherent cell-feeder layers are morphologically dissimilar to mouse BM feeder layers (Hayashi et al., 1984; and unpublished observations). This observation suggests that rat BM-derived microenvironmental cells capable of supporting lymphopoiesis are not supported under the present culture conditions. It is of interest in considering the physiological relevance of our xenogeneic culture system that the mouse BM microenvironment is capable of supporting the development of rat lymphoid cells *in vivo* (Ildstad et al., 1991, 1992) as well as *in vitro*. However, it should be cautioned that, in both instances, the regulation of rat lymphoid cell development by mouse BM stroma may be assisted by the presence of rat-origin microenvironmental cells engrafted along with the hemopoietic cells (Medlock et al., 1987a).

A tentative *in vivo* model for B lymphopoiesis has been proposed in the rat, in which $HIS24^+$ $HIS50^-$ $c\mu^-$ pro-B-cells (1.7% of total nucleated BM cells) give rise to $HIS24^+$ $HIS50^+$ $c\mu^-$ pre-pre-B-cells (5% of total nucleated cells), and then to $HIS24^+$ $HIS50^+$ $c\mu^+$ pre-B-cells (20% of total nucleated cells) (Hermans, 1991). Results in the present study suggest that a similar developmental hierarchy of rat early B-lineage cells exists in our culture system. Moreover, studies in which freshly harvested or culture-generated $HIS24^+$ $HIS50^-$ and $HIS24^+$ $HIS50^+$ rat BM lymphoid cells were separated by flow cytometry and placed in culture indicate that the $HIS50^+$ cells beget mostly $HIS50^+$ cells and have a limited proliferative potential, whereas the $HIS50^-$ cells generate both $HIS50^-$ and $HIS50^+$ cells and proliferate indefintely upon repeated passage (Goldschneider et al., 1989). It is of especial interest therefore that approximately 25% of the lymphoid cells recovered from CM and culture inserts in the present study had an extremely primitive $HIS24^+$ $HIS50^-$ $TdT^-$ $c\mu^-$ B-lineage phenotype, consistent with an even earlier, $B220^+$ $HSA^-$ pre-pro-B-cells, stage of development described in the mouse (Hardy et al., 1991; Tong et al., 1993). The results also suggest that the microenvironmental regulatory requirements for pre-pro-B-cells and pro-B-cells are distinct from those for pre-B-cells. This is consistent with the recent observation (Funk and Witte, 1992) that $c\mu^+$ pre-B-cells and $TdT^+$ pro-B-cells are located in anatomically distinct BM compartments in mice.

The distribution of TdT expression among the HIS50$^+$ and HIS50$^-$ populations of culture-generated lymphoid cells delineates four subsets of lymphoid precursors (Table 1) and permits further insights into the developmental pathway of early B-lineage cells. Thus, although all of the proliferating cells in CM were HIS24$^+$ HIS50$^-$, approximately half were also TdT$^+$ (Table 3). When combined with the demonstration in previous studies that the appearance of TdT$^-$ lymphoid cells precedes that of TdT$^+$ lymphoid cells in our culture system (Medlock et al., 1993b), as well as during ontogeny (Gregoire et al., 1979), the results strongly suggest that the least mature B-lineage cells in the nonadherent phase of our culture system (and the presumptive *in vitro* target of the soluble mediators in CM) are HIS24$^+$ HIS50$^-$ TdT$^-$ pre-pro-B-cells and HIS24$^+$ TdT$^+$ early pro-B-cells. The expression of TdT in the latter cells might then indicate preparation for D-$J_H$ Ig gene rearrangement (Desiderio et al., 1984), the onset of which may be signified by the expression of the HIS50 marker and cessation of cell proliferation at the intermediate pro-B-cells stage. It will be of considerable interest in this respect to determine whether the subset of HIS24$^+$ HIS50$^+$ TdT$^+$ lymphoid cells in our culture system corresponds to that which has undergone partial D-$J_H$ Ig gene rearrangement, whereas the subset of HIS24$^+$ HIS50$^-$ TdT$^+$ lymphoid cells corresponds to that with germline D-$J_H$ configurations (Hunt et al., 1988; Ehlich et al., 1993). If so, preparation for the synthesis of $c\mu$ presumably would follow the cessation of TdT expression in the HIS24$^+$ HIS50$^+$ cells (late pro-B-cells). Such a hypothetical model of the sequence of primitive B-lineage development in our culture system is presented in Fig. 9.

The most mature cells in the prior scheme of lymphopoiesis (HIS24$^+$ HIS50$^+$ TdT$^-$ late pro-B-cells) constitute up to 25% of the total lymphoid cells present in our culture system. Yet $c\mu^+$ pre-B-cells, their presumptive progeny, are produced only sporadically, even in the presence of exogenous IL-7 (unpublished observation). The reason for the failure of these late pro-B-cells to synthesize $c\mu$ *in vitro* is being explored. However, many must be able to do so under the appropriate conditions, because culture-generated cells are approximately 25fold more efficient than are freshly harvested BM cells at producing sIg$^+$ B cells when adoptively transferred *in vivo* (Goldschneider and McKenna, 1991). Furthermore, we have observed that a line of culture-generated intermediate pro-B-cells (HIS24$^+$ HIS50$^-$ TdT$^+$) expressed readily detectible $c\mu$ coincident with spontaneous leukemic transformation *in vitro* (unpublished observations).

Although only the lymphoid progenitor cells in the nonadherent compartment of the culture system were studied in the present experiments, it is likely that many of their counterparts in the adherent compartment are also responsive to the lymphostimulatory activity in CM and that cell contact is optional for most of these cells as well. Thus, we have observed that the lymphoid cells generated in culture inserts or in CM rapidly form foci of adherent lymphopoietic cells when seeded directly onto stromal cell-feeder layers; and that actively cycling lymphoid cells from the adherent and nonadherent phases of the culture system constitute phenotypically indistinguishable subpopulations (Hayashi et al., 1984; Medlock et al., 1993b). Nonetheless, it is possible that the most primitive lymphoid precursors in these cultures require cell contact in addition to other signals for optimal stimulation, as suggested by the report of Hardy et al. (1991) for pre-pro-B-cells in the mouse.

To our knowledge, the present study is the first to demonstrate that populations of committed lymphoid stem/progenitor cells, devoid of detectible pluripotent hemopoietic stem cells (Hayashi et al., 1984; and unpublished observations), can be generated for prolonged periods in culture inserts. Such cell populations appear to include prothymocytes as well as pro-B-cells, as demonstrated by *in vivo* adoptive transfer studies. Parallel experiments using medium conditioned by stromal cell lines from mouse BM suggest that this early lymphopoietic activity is maintained by one or more stage-specific soluble mediators. The precise nature of this lymphostimulatory activity is presently being investigated. However, preliminary experiments indicate that the major factor is a novel high MW form of IL-7 that is bound, but not neutralized, by antibodies to IL-7.

### MATERIALS AND METHODS

### Animals

Male 4–6-week-old C57BL/6 strain mice, purchased from the National Cancer Institute (NCI) (Frederick, MD), were used as donors of BM adherent cells. Male 4–6-week-old Lewis strain rats, bred from

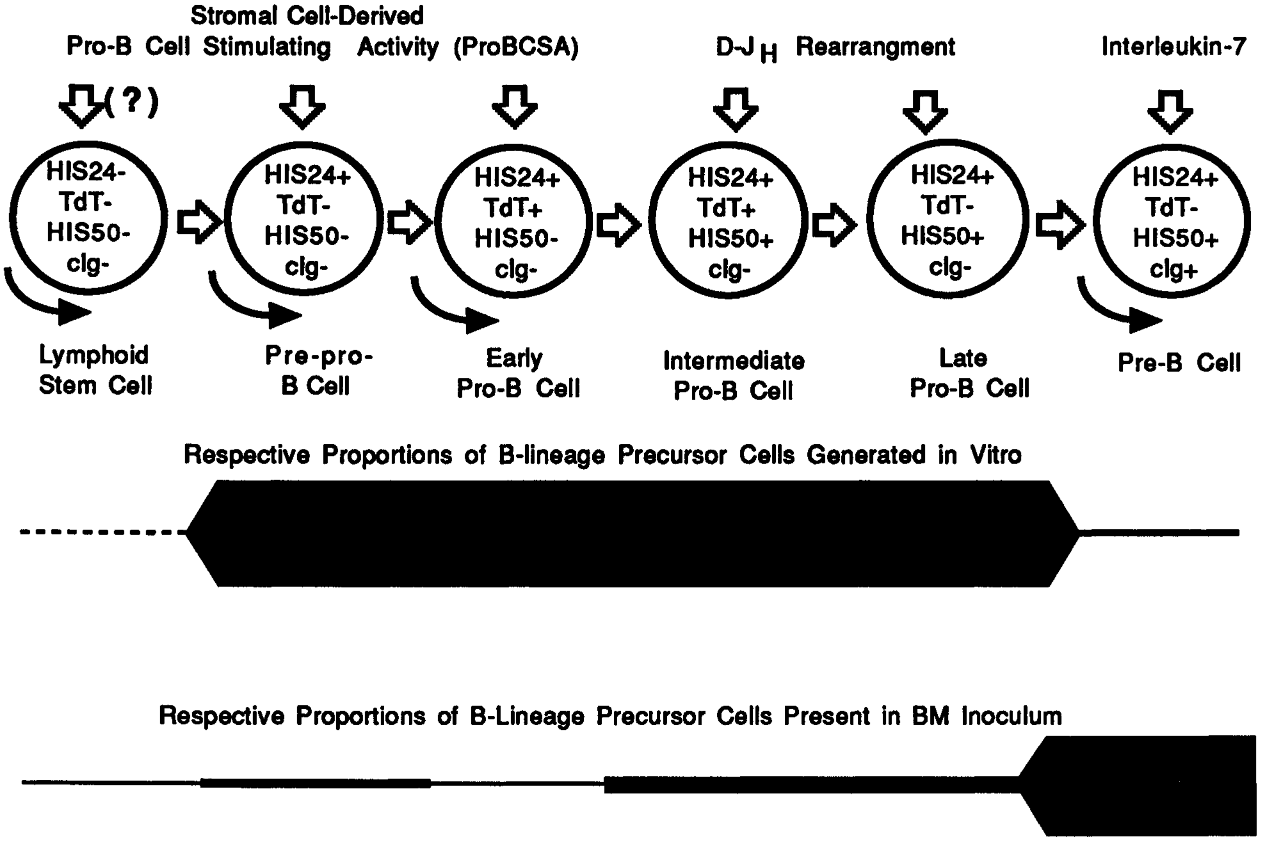

FIGURE 9. Hypothetical scheme of early B-lineage development in long-term xenogeneic cultures of rat BM cells on mouse BM-adherent cell-feeder layers or conditioned medium. Solid bars delineate lymphoid cell subsets routinely generated in culture, as compared with those present in the original B inoculum. Horizontal straight arrows indicate probable parent–progeny relationships of phenotypically defined cell subsets. Vertical straight arrows indicate probable stage of development at which indicated events occur. Curved arrows designate probable stages of development at which cell proliferation occurs. It is not known if ProBCSA, which stimulates proliferation of pre-pro-B and early pro-B-cells, also regulates the differentiation of intermediate and/or late pro-B-cells.

stock originally obtained from the NCI, were used as donors of BM lymphoid precursor cells.

### Antibodies

Murine monoclonal antibody (mAb) to the mouse MHC alloantigen, H-$2^b$, was used as the supernatant of the 28-8-6S cell line (Ozato and Sachs, 1981) obtained from the American Type Culture Collection (ATCC) (Rockville, MD). Alloantiserum to the rat MHC alloantigen, RT-$1^l$, was prepared by immunizing M520 strain rats (RT-$1^b$) with Lewis rat (RT-$1^l$) lymph node cells as described (Lubaroff and Waksman, 1968). HIS40 (anti-IgM) (Deenen et al., 1987), HIS24 (anti-CD45R-B220) (Deenen et al., 1987; Kroese et al., 1987), and HIS50 (anti-Heat Stable Antigen/HSA) (Hermans, 1991; Tong et al., 1993) murine mAb to rat B-lineage associated antigens were generously provided by Dr. Davine Opstelten, Department of Pathology, University of Hong Kong. OX19 (pan-rat T cell) mAb was purchased from Accurate Chemical and Scientific Corp. (Westbury, NY). Mouse monoclonal antibodies to rat Igk-$1^a$ (MAR 80.2) and Igk-$1^b$ (RG 11/15.1) light-chain allotypes, expressed on the surface of 95% of rat B cells (Springer et al., 1982), were the gift of Dr. G. A. Gutman (University of California, Irvine) (Gutman, 1982; Lanier et al., 1982). Rat monoclonal antibodies to RT-$7^a$ (BC84) and RT-$7^b$ (8G6.1) rat pan-T cell alloantigens were gifts from Dr. D. M. Lubaroff (University of Iowa, Iowa City) (Ely, et al., 1983). Mouse anti-bromodeoxyuridine (anti-BrdU) mAb (with nuclease) was purchased from Amersham International (Amersham, England).

Affinity-purified fluorescein isothiocyanate (FITC)-conjugated goat IgG F(ab')$_2$ anti-mouse and anti-rat IgG antibodies, and FITC-conjugated goat anti-mouse IgM (heavy-chain-specific) antibody were obtained from Kirkegaard and Perry Laboratories (Gaithersburg, MD). Affinity-purified rabbit antibody to calf thymus terminal deoxynucleotidyl transferase (TdT), and FITC-and tetramethylrhodamine isothiocyanate (TRITC)-conjugated goat anti-rabbit IgG were purchased from Supertechs (Bethesda, MD). Phycoerythrin (PE)-conjugated goat anti-mouse IgG was obtained from Caltag Laboratories (San Francisco).

### Immunofluorescence

Indirect immunofluorescence of cell-surface antigens was performed by incubating $1 \times 10^6$ freshly harvested or culture-generated cells with mouse or rat primary antibodies (10 $\mu$l) and developing with appropriate FITC-or PE-conjugated goat anti-IgG or IgM antibodies (Hayashi, et al., 1984). To detect intranuclear TdT, cytocentrifuge-prepared cell smears were fixed in 4°C absolute methanol, stained with rabbit antibodies to TdT, and developed with FITC- or TRITC-conjugated antibodies to rabbit IgG (Gregoire et al., 1977). Double immunofluorescence for surface or cytoplasmic $\mu$ Ig heavy chains and TdT was performed as described (Goldschneider et al., 1987). Briefly, cytocentrifuge smears were fixed in cold absolute ethanol with 5% glacial acetic acid for 20 min at 4°C, stained for TdT as described before, and developed with TRITC-goat anti-rabbit IgG. The slides were then washed, stained with HIS40 mAb, and developed with FITC-goat anti-mouse IgG. Labeled cells were quantified using a Zeiss universal fluorescence microscope equipped with narrow-band filters for fluorescein and rhodamine.

To detect the incorporation of BrdU, cultured cells were pulsed overnight with BrdU cell-proliferation labeling reagent (Amersham International) in a final concentration of 1:1000. Cytosmears prepared from these cells were fixed in cold absolute ethanol with 5% glacial acetic acid, stained with the anti-BrdU/nuclease reaction mixture for 60 min, and developed with FITC-goat anti-mouse IgG. Double immunofluorescence for BrdU and TdT was accomplished by staining for TdT at this step. Double immunofluorescence for BrdU and cell-surface antigens was performed by staining cells in suspension with antibodies to surface antigens as described before, and then staining cytocentifuge smears of the same cells for BrdU.

### Cell-Culture System

Rat BM lymphoid precursor cells were generated in our xenogeneic culture system as previously described (Hayashi and Goldschneider, 1982; Hayashi et al., 1984). Briefly, adherent cell-feeder layers were established by asceptically flushing BM from the femura of mice with RPMI-1640. Single cell suspensions made therefrom were added to 2 ml RPMI-1640 containing 25% lot-selected, defined FBS (Hyclone, Logan, UT) in 35-mm-diameter culture plate wells ($8 \times 10^6$ cells/well) and incubated at 37°C in 5% $CO_2$. After 10 days, the confluent feeder layers were washed with RPMI-1640 and seeded with $1 \times 10^6$ freshly harvested rat BM cells. In some experiments, the rat BM cells were seeded into microporous membrane culture inserts (0.4 $\mu$m pore size) placed over (but not in contact with) the mouse BM feeder layers. Both Transwell #3408 (Costar, Cambridge, MA) and Millicell-HA (Millipore Corp., Bedford, MA) insert units were found to be suitable. In other experiments, both the mouse BM feeder layers and the culture inserts were seeded with $1 \times 10^6$ freshly harvested rat BM cells. Total cells from the culture inserts and nonadherent lymphoid cells from the standard cultures were recovered on day 10 for cytologic and phenotypic analysis or for transfer to secondary culture inserts and/or mouse BM adherent cell feeder layers.

### Conditioned Medium

Day 10 adherent cell-feeder layers from mouse BM, spleen, and thymus were washed and refed with 2 ml/well fresh culture medium containing 25% FBS. After 10 additional days of culture, the conditioned medium (CM) was recovered, centrifuged to remove cells, and sterilized by passage through a 0.22-$\mu$m Millex-GV filter (Millipore Corp.). In some experiments, the CM was concentrated by ultrafiltration in Centriprep-10 Concentrator units (Amicon, Danvers, MA) or an Amicon stirred cell-filtration system.

Freshly harvested rat BM cells or cells obtained on day 10 from standard cultures or culture inserts were added to wells ($5 \times 10^5$ cells/well) containing 2 ml of concentrated or unconcentrated CM of feeder layers. At various times thereafter, cells were recovered for phenotypic analysis and/or secondary transfer. To evaluate cell proliferation, nonadherent rat BM lymphoid cells harvested on day 10 from standard cultures were washed and cultured ($1 \times 10^5$ cells/well) in CM or control medium for 3 days in Costar 96-well, flat-bottomed culture plates. The cells were then pulsed with 1 $\mu$Ci/well of [$^3$H]TdR (New England Nuclear, Boston) 12 hr prior to harvesting. Incorporation of [$^3$H]TdR was determined by liquid scintillation spectroscopy.

### *In Vivo* T- and B-Cell Regeneration Assay

To examine whether CM-sensitive rat BM cells were capable of regenerating the T- and B-cell compartments *in vivo*, freshly harvested Albany strain rat BM cells were cultured in T-75 culture flasks containing unconcentrated mouse BM adherent cell CM or control medium ($1 \times 10^6$ cells/ml) for 4 days. Cells were then harvested, washed extensively with serum-free RPMI, and suspended at $10 \times 10^6$ cells/ml in RPMI. One milliliter of cell suspension was injected into the lateral tail vein of each irradiated (600 rads) M520 strain recipient rat. After 25 days, the rats were sacrificed and the thymus and spleens collected. Single cell suspensions from chimeric as well as medium-injected control rats were stained by immunofluorescence for appropriate donor and host specific T- and B-cell markers.

### Establishment of Mouse BM Stromal Cell Lines

Day 10 primary mouse BM adherent cell-feeder layers grown in RPMI-1640 with 25% FBS were extensively washed, detached with 0.1% trypsin (Gibco Laboratories, Grand Island, NY), and dissociated by gentle pippetting. The suspended cells were washed in 25% FBS-containing culture medium, plated in 35-mm culture plate wells ($1 \times 10^6$ cells/well), and grown to confluency. The adherent cell layers were then washed, fed with fresh medium, and cultured for an additional 10 days. At this time, the medium was tested for its ability to maintain culture-generated rat BM lymphoid precursor cells as before, and adherent cell layers with such activity were selected for further passaging at 10-day intervals. This process was repeated for approximately 2 months by which time biologically active, morphologically homogenous stromal cell lines were established.

### ACKNOWLEDGMENTS

This work was supported in part by grants GM-38306 from the National Institutes of Health and IM-645 from the American Cancer Society.

*(Received January 29, 1993)*

*(Accepted May 12, 1993)*

### REFERENCES

Billips L.G., Petitte D., Dorshkind K., Narayanan R., Chiu C.P., and Landreth K.S. (1992). Differential roles of stromal cells, interleukin-7, and kit-ligand in the regulation of B lymphopoiesis. Blood **79:** 1185–1192.

Deenen G.J., Hunt S.V., and Opstelten D. (1987). A stathmokinetic study of B lymphocytopoiesis in rat bone marrow: Proliferation of cells containing cytoplasmic u-chains, terminal deoxynucleotidyl transferase and carrying HIS24 antigen. J. Immunol. **139:** 702–710.

Denis K.A., Dorshkind K., and Witte O.N. (1987). Regulated progression of B lymphocyte differentiation from cultured fetal bone marrow. J. Exp. Med. **166:** 391–403.

Denis K.A., Treiman L., St. Clair J., and Witte O.N. (1984). Long-term cultures of murine fetal liver retain very early B lymphoid phenotype. J. Exp. Med. **160:** 1087–1095.

Desiderio S.V., Yancolopoulos G.D., Paskind M., Thomas E., Boss M., Landau N., Alt F.W., and Baltimore D. (1984). Insertion of N region into heavy-chain genes is correlated with the expression of terminal deoxytransferase in B cells. Nature **311:** 752–755.

Dexter T.M., Allen T.D., and Lathja L.G. (1977). Conditions controlling the proliferation of haemopoietic stem cells *in vitro*. J. Cell Physiol **91:** 335–344.

Dorshkind K. (1990). Regulation of hemopoiesis by bone marrow stromal cells and their products. Ann. Rev. Immunol. **8:** 111–137.

Ehlich A., Schaal S., Gu H., Kitamura D., Müller W., and Rajewsky K. (1993). Immunoglobulin heavy and light chain genes rearrange independently at early stages of B cell development. Cell **72:** 695–704.

Ely J.M., Greiner D.L., Lubaroff D.M., and Fitch F.W. (1983). Characterization of monoclonal antibodies that define rat T cell alloantigens. J. Immunol. **130:** 2798–2803.

Flanagan J.G., and Leder P. (1990). The kit ligand: A cell surface molecule altered in steel mutant fibroblasts. Cell **63:** 185–194.

Funk P.E., and Witte P.L. (1992). Enrichment of primary lymphocyte-supporting stromal cells and characterization of associated B lymphocyte progenitors. Eur. J. Immunol. **22:** 1305–1313.

Goldschneider I., and King T. (1991). *In vitro* cultivation of normal and leukemic lymphoid precursors from human bone marrow. FASEB J. **5:** A1001.

Goldschneider I., and McKenna S.D. (1991). Generation of primitive rat lymphoid precursor cells *in vitro* is mediated by a soluble factor(s) derived from mouse BM stromal cells. J. Cell. Biochem. **Suppl. 15F:** 50.

Goldschneider I., Medlock E.S., Opstelten D., and Greiner D.L. (1987). B lymphocyte-associated HIS antibodies delineate subsets of rat bone marrow-derived TdT$^+$ cells generated *in vitro* Transplant. Proc. **19:** 3161–3165.

Goldschneider I., Opstelten D., and Hermans M. (1989). Generation of two subsets of TdT$^-$ positive pre-pre-B-cells from rat bone marrow in a long-term culture system. *In*: Progress in immunology VII, Melchers F., et al. (New York: springer-Verlag), p. 185, Abstract 35–9.
Gordon M.Y., Riley G.P., Watt S.M., and Greaves M.F. (1987). Compartmentalization of a haemopoietic growth factor (GM-CSF) by glycosaminoglycans in the bone marrow miroenvironment. Nature **326:** 403–405.
Gregoire K.E., Goldschneider I., Barton R.W., and Bollum F.J. (1977). Intracellular distribution of terminal deoxynucleotidyl transferase in rat bone marrow and thymus. Proc. Natl. Acad. Sci. USA **74:** 3993–3996.
Gregoire K.E., Goldschneider I., Barton R.W., and Bollum F.J. (1979). Ontogeny of terminal deoxynucleotidyl-transferase-positive cells in lymphohemopoietic tissues of rat and mouse. J. Immunol. **123:** 1347–1352.
Gutman G.A. (1982). Rat kappa chain allotyptes. IV. Monoclonal antibodies to distinct RI-1b specificities. Hybridoma **1:** 133–139.
Hardy R.R., Carmack C.E., Shinton S.A., Kemp J.D., and Hayakawa K. (1991). Resolution and characterization of pro-B and pre-pro-B-cell stages in normal mouse bone marrow. J. Exp. Med. **173:** 1213–1225.
Hayashi J., and Goldschneider I. (1982). *In vitro* culture of rat bone-marrow cells positive for terminal deoxynucleotidyl transferase. *In*: cold spring Harbor conferences on cell proliferation, Vol. 9, Sato G.H., et al., Eds. (Cold Spring Harbor, NY: Cold Spring Harbor Press), pp. 665–675.
Hayashi J., Medlock E.S., and Goldschneider I. (1984). A selective culture system for generating terminal deoxynucleotidyl transferase-positive lymphoid precursor cells *in vitro*. I. Description of the culture system. J. Exp. Med. **160:** 1622–1639.
Henney C.S. (1989). Interleukin 7: Effects on early events in lymphopoiesis. Immunol. Today **10:** 170–173.
Hermans M. (1991). B lymphocyte development in rat bone marrow (Hong Kong: United League Graphic and Printing Co.).
Huang E., Nocka K., Beier D.R., Chu T.Y., Buck J., Lahm H.W., Wellner, D., Leder P., and Besmer P. (1990). The hemopoietic growth factor KL is encoded by the S1 locus and is the ligand of the c-kit receptor, the gene product of the W locus. Cell **63:** 225–233.
Hunt S.V., Medlock E.S., Greiner D.L., Goldschneider I., and Opstelten D. (1988). Rat immunoglobin genes have comparable patterns of JH rearrangements in normal peripheral B-cells and in pre-B and cultured TdT$^+$ cells from bone marrow. Adv. Exp. Med. Biol. **237:** 63–68.
Ildstad S.T., Vacchio M.S., Markus P.M., Hronakes M.L., Wren S.M., and Hodes R.J. (1992). Cross-species transplantation tolerance: Rat bone marrow-derived cells can contribute to the ligand for negative selection mouse T cell receptor V$\beta$ in chimeras tolerant to xenogeneic antigens (mouse+rat → mouse). J. Exp. Med. **175:** 145–155.
Ildstad S.T., Wren S.M., Boggs S.S., Hronakes M.L., Vecchini F., and Van den Brink M.R.M. (1991). Cross-species bone marrow transplantation: Evidence for tolerance induction, stem cell engraftment, and maturation of T lymphocytes in a xenogeneic stromal environment (rat → mouse). J. Exp. Med. **174:** 467–478.
Kierney P., and dorshkind K. (1987). B. lymphocyte precursor cells and myeloid progenitors survive in diffusion chamber cultures but B-cell differentiation requires close association with stromal cells. Blood **70:** 1418–1424.
Kincade P.W., Lee G., Pietrangeli C.E., Hayashi S.I., and Gimble J.M. (1989). Cells and molecules that regulate B lymphopoiesis in bone marrow. Ann. rev. Immunol. **7:** 111–143.
Kroese F.G.M., Wubbena A.Ṡ., Opselten D., Deenen G.J., Schwander E.H., De Leij L., Vos H., Poppema S., Volberda J., and Nieuwenhuis P. (1987). B lymphocyte differentiation in the rat: Production of monoclonal antibodies to B lineage-associated antigens. Eur. J. Immunol. **17:** 921–928.
Lanier L.L., Gutman G.A., Lewis D.E., Griswold S.T., and Warner N.L. (1982). Monoclonal antibodies against rat immunoglobulin kappa chains. Hybridoma **1:** 125–132.
Lee G., Namen A.E., Gillis S., Ellingsworth L.R., and Kincade P.W. (1989). Normal B cell precursors responsive to recombinant murine IL-7 and inhibition of IL-7 activity by transforming growth factor-B. J. Immunol. **142:** 3875–3883.
Lubaroff D.M., and Waksman B.H. (1968). Bone marrow as a source of cells in reactions of cell hypersensitivity. II. Identification of allogeneic or hybrid cells by immunofluorescence in passively transformed tuberculin reactions. J. Exp. Med. **128:** 1437–1447.
Matuoka K., Masayoshi N., and Mitsui Y. (1987). Hyaluronate synthetase inhibition by normal and transformed human fibroblasts during growth reduction. J. Cell Biol. **104:** 1105–1115.
McNiece I.K., Langley K.E., and Zsebo K.M. (1991). The role of recombinant rat-derived stem cell factor in early B-cell development. Synergistic interaction with IL-7. J. Immunol. **146:** 3785–3790.
Medlock E.S., Goldschneider I., and Greiner D.L. (1987a). A selective culture system for generating terminal deoxynucleotidyl transferase-positive lymphoid cells *in vitro*. II. A rat bone marrow accessory cell promotes the growth of mouse bone marrow TdT$^+$ cells. Transplant. Proc. **19:** 3175–3178.
Medlock E.S., Goldschneider I., Greiner D.L., and Shultz L. (1987b). Defective lymphopoiesis in the bone marrow of motheaten (me/me) and viable motheaten (me$^v$/me$^v$) mutant mice. II. Description of a microenviromental defect for the generation of terminal dexoynucleotidyl transferase-positive bone marrow cells *in vitro*. J. Immunol. **138:** 3590–3597.
Medlock E.S., McKenna S.D., and Goldschneider I. (1993a). A selective culture system for generating terminal dexoynucleotidyl transferase-positive lymphoid cells *in vitro*. III. Interaction of lymphoid progenitor cells with the stromal cell microenvironment. Lab. Invest. In press.
Medlock E.S., McKenna S.D., and Goldschneider I. (1993b). A selective culture system for generating terminal deoxynucleotidyl transferase-positive lymphoid cells *in vitro*. IV. Properties of the lymphoid cells in the adherent and non-adherent phases of the culture. Lab. Invest. In press.
Miyake K., Medina K.L., Hayashi S.I., Ono S., Hamaoka T., and Kincade P.W. (1990). Monoclonal antibodies to Pgp-1/CD44 block lympho-hemopoiesis in long-term bone marrow cultures. J. Exp. Med. **171:** 477–488.
Namen A.E., Lupton S., Hjerrild K., Wignall J., Mochizuki D.Y., Schmierer A., Mosley B., March C.J., Urdal D., Gillis S., Cosman D., and Goodwin R.G. (1988a). Stimulation of B-cell progenitors by cloned murine interleukin-7. Nature **333:** 571–573.
Namen A.E., Schmierer A.E., March C.J., Overell R.W., Park L.S., Urdal D.L., and Mochizuki D.Y. (1988b). B-cell precursor gowth-promoting activity. Purification and characterization of a growth factor on lymphoid precursors. J. Exp. Med. **167:** 988–1002.
Nishikawa S.I., Ogawa M., Nishikawa S., Kunisada T., and Kodama H. (1988). B lymphopoiesis on stromal cell clone: stromal cell clones acting on different stages of B cell differentiation. Eur. J. Immunol. **18:** 1767–1771.
Opstelten D., Deenen G.J., Rozing J., and Hunt S.V. (1968). B lymphocyte-associated antigens on terminal deoxynucleotidyl transferase-positive cells and pre-B-cells in bone marrow of the rat. J. Immunol. **137:** 76–84
Ozato K., and Sachs D.H. (1981). Monoclonal antibodies to murine mouse MHC antigens. II. Hybridoma antibodies reacting to antigens of the H-2b haplotype reveal genetic control of isotype expression. J. Immunol. **126:** 317–321.
Peschel C., Green I., and Paul W.E. (1989). Preferential proliferation of immature B lineage cells in long-term stromal celldependent cultures with IL-4. J. Immunol. **142:** 1558–1568.

Roberts R., Gallagher J., Spooncer E., Allen T.D., Bloomfield F., and Dexter T.M. (1988). Heparan sulfate bound growth factors: A mechanism for stromal cell mediated haemopoiesis. Nature **332:** 376–378.

Scherele P.A., Dorshkind K., and Witte O.N. (1990). Clonal lymphoid progenitor cell lines expressing the BCR/ABL oncogene retain full differential function. Proc. Natl. Acad. Sci. USA **87:** 1908–1912.

Schrader J.W., Goldschneider I., Bollum F.J., and Schrader S. (1978). *In vitro* studies in lymphocyte differentiation. II. Generation of terminal dexoynucleotidyl transferase-positive cells in long-term cultures of mouse bone marrow J. Immunol. **122:** 2337–2343.

Springer T.A., Bhattacharya A., Cordoza J.T., and Sanchez-Madrid E. (1982). Monoclonal antibodies specific for rat IgG1, IgG2a and IgG2b subclasses and kapa chain monotypic and allotypic determinants, reagents for use with monoclonal antibodies. Hybridoma **1:** 257–264.

Sudo T., Ito M., Ogawa Y., Iizuka M., Kodama H., Kunisada T., Hayashi S.I., Ogawa M., Sakai K., Nishikawa S., and Nishikawa S.I. (1989). Interleukin 7 production and function in stromal cell-dependent B-cell development. J. Exp. Med. **170:** 333–338.

Tong T., Hunt S.V., and Opstelten D. (1993). HIS50, a rat homolog to mouse J11d/Heat stable antigen, and human CD24; cDNA isolation and sequence. J. Cell. Biochem. **Suppl. 17B;** 242.

Whitlock C.A., Robertson D., and Witte O.N. (1984). Murine B-cell lymphopoiesis in long term culture. J. Immunol. Methods **67:** 353–369.

Whitlock C.A., and Witte O.N. (1982). Long term culture of B lymphocytes and their precursors from murine bone marrow. Proc. Natl. Acad. Sci USA **79:** 3608–3612.

Williams D.E., Eisenman J., Baird A., Rauch C., Van Ness K., March C.J., Park L.S., Martin U., Mochizuki D.Y., Boswell H.S., Burgess G.S., Cosman D., and Lyman S.D. (1990). Identification of a ligand for the c-kit proto-oncogene. Cell **63:** 167–174.

Zsebo K.M., Wypych J., McNiece I.K., Lu H.S., Smith K.A., Karkare S.B., Sachdev R.K., Yuschenkoff V.N., Birkett N.C., Williams L.R., Satyagal V.N., Tung W., Bosselman R.A., Mendiaz E.A., and Langley K.E. (1990). Identification, purification, and biological characterization of hemopoietic stem cell factor from Buffalo rat liver conditioned medium. Cell **63:** 195–201.

